# Laminar airflow ventilation systems in orthopaedic operating room do not prevent surgical site infections: a systematic review and meta-analysis

**DOI:** 10.1186/s13018-023-03992-2

**Published:** 2023-08-05

**Authors:** Xueqian Ouyang, Qiaolin Wang, Xiaohua Li, Ting Zhang, Sanjay Rastogi

**Affiliations:** 1 Anesthesia operating room, Xianyang Central Hospital, 712000 Xianyang, People’s Republic of China; 2 Department of Internal Medicine Ward I, Yantai Qishan Hospital, Yantai, 264001 Shandong Province People’s Republic of China; 3 Vasculocardiology Department, Xianyang Central Hospital, Xianyang, 712000 People’s Republic of China; 4 Anesthesia operating room, Northwest Women’s and Children’s Hospital, 710061 Xi’an, People’s Republic of China; 5 ESIC Model Hospital, ESIC, Pir Ajan Fakir Rd, Guwahati, 781021 Assam India

**Keywords:** HEPA filters, Laminar air flow, Meta-analysis, Orthopaedic surgeries, Operating rooms, Surgical site infections, Wound healing

## Abstract

**Background:**

Laminar airflow (LAF) technologies minimize infectious microorganisms to enhance air quality and surgical site infections (SSIs). LAF lowers SSIs in some clinical studies but not others. This study analyzes laminar airflow ventilation's capacity to reduce orthopaedic surgery-related SSIs.

**Methods:**

The PRISMA-compliant keywords were utilized to conduct a search for pertinent articles in various databases including PubMed, MEDLINE, CENTRAL, Web of Sciences, and the Cochrane databases. Observational studies, including retrospective, prospective, and cohort designs, satisfy the PICOS criteria for research methodology. The assessment of quality was conducted utilizing the Robvis software, while the meta-analysis was performed using the RevMan application. The study’s results were assessed based on effect sizes of odds ratio (OR) and risk ratio (RR).

**Results:**

From 2000 to 2022, 10 randomized controlled clinical trials with 10,06,587 orthopaedic surgery patients met the inclusion criteria. The primary outcomes were: (1) Risk of SSI, (2) Bacterial count in sampled air and (3) Reduction in SSIs. The overall pooled OR of all included studies was 1.70 (95% CI 1.10–2.64), and the overall pooled RR was 1.27 (95% CI 1.02–1.59) with p < 0.05. LAF is ineffective at preventing SSIs in orthopaedic procedures due to its high-risk ratio and odds ratio.

**Conclusions:**

The present meta-analysis has determined that the implementation of LAF systems does not result in a significant reduction in the incidence of surgical site infections (SSIs), bacterial count in the air, or SSIs occurrence in orthopaedic operating rooms. Consequently, the installation of said equipment in operating rooms has been found to be both expensive and inefficient.

## Introduction

These days, cardiac, orthopaedic, brain, ophthalmology, and other surgical procedures are frequent, although their purposes vary. They repair, remove, and reposition the injured tissues, organs, and blockages [1, 2]. Surgery is risky due to direct intraoperative trauma, perioperative infections, hematoma development, and postoperative infection [3, 4]. Staphylococcus, Streptococcus, and Pseudomonas bacteria cause most surgical site infections [5, 6]. These infections might be mild or severe, affecting the skin, tissues, organs, or biomaterial inserted during surgery [7].

Infections cause shortness of breath, confusion, acute discomfort, shivering, fever, a fast heart rate, and disorientation [8, 9]. These conditions may be fatal if left untreated. Thus, mortality is 0.4% and morbidity is 3–17% among the 250 million procedures conducted worldwide [10, 11]. Thus, to prevent these infections from affecting patients' health following surgery, the operating room must be sterile. This can be achieved with the use of scrub suits, clean air suits, sterile dressings, and a laminar airflow ventilation system (LAF) [12]. LAF through filtration equipment creates an ultraclean zone around the operation site [13].

LAF systems are useful for maintaining sterile conditions in the operating room because their microbial sedimentation plates produce a continuous flow of microorganism-free air, which improves air quality by reducing infectious microbes [14]. In their review article, James et al. [15] found that LAF theatres reduce microorganisms in operating theatre air. In their systematic review and meta-analysis, Liu et al. [16] found that LAF systems minimise surgical site infections (SSIs) by eliminating airborne germs.

Despite ample correlations linking LAF to reduced rates of surgical site infection, numerous studies have found it to be ineffective at lowering infection rates in the operating room during orthopaedic procedures. For instance, Friberg et al. [17] reported that the use of horizontal LAF units is seriously questionable, while Kakwani et al. [18] suggested the use of LAF in operating theatres. While Brandt et al. [19] reported that the use of LAF in operating theatres is highly risky and, instead of reducing it, it increases the chances of infection.

On the other hand, Nilson et al. [20] support its use in reducing SSIs. However, Sossai et al. [21], Hooper et al. [22] do not mention its use. Similarly, Bosanquet et al. [23] found its installation worthwhile for vascular surgery while Pinder et al. [24], Wang et al. [25], and Langvatan et al. [26] mentioned that installation of LAF is not suitable in operating theatres as it increases the chance of SSIs and unnecessary increases the financial burden owing to its high installation cost.

As there are contradictory studies regarding the use of LAF in the operating theatre, we systematically reviewed and meta-analysed the different studies on the role of LAF systems in the prevention of SSIs in operating rooms to evaluate the benefits and efficacy of LAF.

## Material and methods

We followed the guidelines of PRISMA normative recommendations [27] in the present study with the registration number XCH#/IRB/2022/986.

### Search strategy

This meta-analysis is based on an extensive search conducted in the databases of Medline (via PubMed), Cinahl (via Ebsco), Scopus, and WoS from the year 2000 till 2022.An inclusive literature search was conducted without any limitations on the year and language of publication utilizing the electronic databases Cochrane Library, EMBASE, and PubMed using the following search criteria: (I) “laminar airflow”; OR LAF; (II) “Surgical site infections” OR SSI; (III) “ High Efficiency Particulate Air Filter” OR & HEPA filters; (IV) “ Reduction in risk of SSIs”; (V) “Bacterial count in sampled air of operating rooms”; (VI) “wound healing”; (VII) “Orthopedic surgery”; (VIII) “sterile conditions” and (IX) “ Post-operative infections”. Within the context of the search strategy, the Boolean operator “AND” was used to combine the Medical Subject Headings (MeSH) with the text keywords. First, duplicate articles were deleted from the search results, followed by a title and abstract screening of the remaining articles. Finally, the full texts of all the qualified studies were retrieved and reviewed for inclusion and exclusion based on the inclusion—exclusion criteria. The full-text articles of the sources were collected and abstracts were used only if they had sufficient information for the meta-analysis. Articles were included following the PRISMA guidelines and studies were selected randomly as per the PICOS criteria as shown in Table 1, irrespective of the type of study (randomized clinical trial, comparative study, prospective study, or retrospective study). Two authors **(**XO and QW**)** separately scanned the relevant sources for related studies. A demographic summary of the patients and event data with useful variables was extracted from the included studies [17–26] by two researchers (XL and TZ) independently.

**Table 1:** PICOS Search

P (patient, problem, population)	Patient underwent orthopaedic surgeries
I (intervention)	Evaluation of effect of laminar airflow ventilation system in the prevention of surgical site infections (SSIs) in operating room
C (comparison, control or comparator)	Comparison of operating rooms with or without laminar air flow
O [outcome (s)]	laminar airflow ventilation system is not effective in the prevention of surgical site infections (SSIs) in operating room
S (study type)	Randomized controlled trials, cohort study, comparative study, prospective study, retrospective study

### Inclusion and exclusion criteria

Those studies included those that reported the use of a LAF ventilation system in an operating theatre for preventing SSIs and its comparison with other conventional filtration systems. Studies were selected from the years 2000 to 2022. In the present study, we only selected studies with the full text and sufficient data for a 2 × 2 table, while abstracts, studies with insufficient data, and related studies published before 2000 were excluded.

### Evaluation of the analytical standard and source of heterogeneity

The methodological validity of the included studies was separately evaluated by two reviewers **(**XO and QW), and the heterogeneity of the included experiments was calculated. Author TZ was responsible for resolving any type of disagreement between authors (XO and QW**)**. The heterogeneity was investigated by Cochran statistics, and the I^2^ index in random bivariate mode was calculated with the help of RevMan software [28] and MedCalc software [29]. The investigated heterogeneity sources were the use of randomized controlled trials vs. comparative studies; retrospective vs. prospective studies, different numbers of patients undergoing surgery; different types of surgery, and the use of different filtration systems.

### Evaluation of risk of bias

The Robvis tool [30] was used to assess the quality of included studies and risk of bias graph and risk of bias summary was designed. This table documented random sequence generation, allocation concealment, blinding of participants and personnel, blinding of outcome assessment, insufficient outcome data, selective reporting, and other forms of bias. We were able to assign a score of low; high, critical and serious to each parameter in our quality assessment of the study using this table. The inquiry was conducted independently by two distinct investigators (LYN and LQP), and the subsequent disagreement was addressed by a third investigator (WX). Publication bias was assessed by Begg's test, Egger’s test, and Deek’s funnel plot [30] via MedCalc software.

### Statistical analysis

A meta-analysis was performed by RevMan and MedCalc software. For statistical analysis, the diagnostic odds ratio and risk ratio were calculated by the DerSimonian Lair technique using a 2 X 2 table made with the help of the event data. Statistical parameters like odds ratio and risk difference were calculated and their respective forest plots were plotted using RevMan software. The heterogeneity of studies was evaluated in terms of chi^2^ value, tau^2^ value, df value, I^2^ value, z-value, and p-value.

## Results

### Literature search results

**Fig.1:**
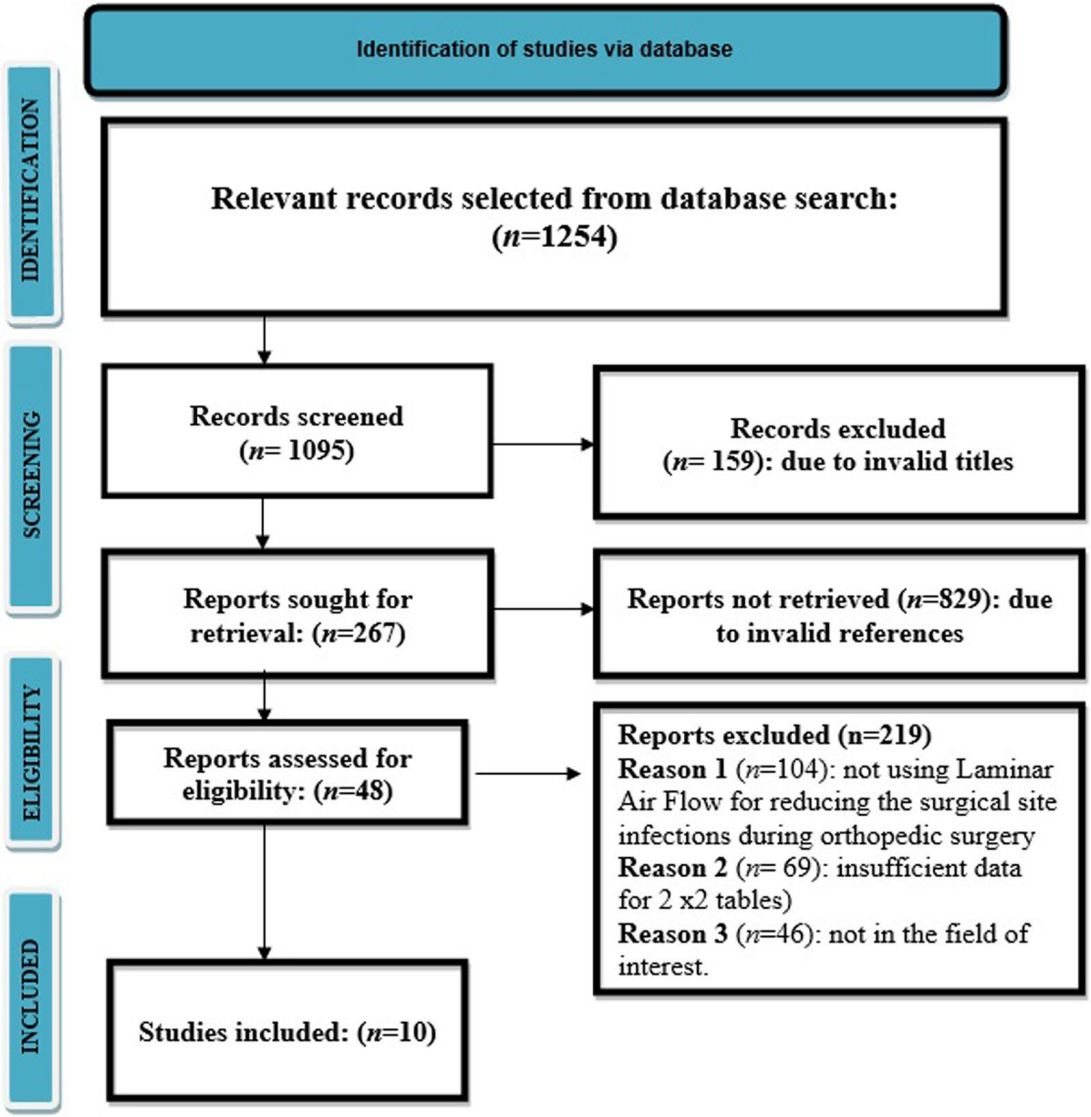
PRSIMA flow diagram of the included studies

We found a total of 1254 studies through electronic scans from different databases as per the PICOS criteria [31]. Among these studies, we excluded 159 studies by reading their titles and abstracts, and 1095 records were screened. Further, due to invalid references and duplicity, we excluded 829 studies and included only 267 studies for final screening. Out of these 267 studies, 219 studies were excluded based on the inclusion criteria, and the eligibility of the remaining 48 studies was assessed further. The key reasons for omission were inadequate evidence and inappropriate comparison criteria to create 2 × 2 tables for review. Finally, for meta-analysis, 10 studies ranging from the years 2000 to 2022 that fulfil the inclusion criteria, i.e., the use of LAF in operation theatres during orthopaedic surgeries for reducing SSIs were used as shown in Fig. 1**.** Included studies reported a total of 10,06,587 patients who underwent surgery. These patients underwent surgery in operating theatres that were either equipped with LAF or not. The descriptive details of the studies included in this meta-analysis are shown in Table 2. It describes the author of the study, publishing year, type of study, the intervention of the study, the total number of surgical departments, total number of surgeries, types of ventilators used in different departments, outcomes, the conclusion of the study, and p-value. Later, this event data was used to perform the meta-analysis.

**Table 2:** Brief summary of the Included studies

Study Id and Year	Journal of publication	Type of study	Total number of surgical departments	Total number of orthopedic surgeries	Intervention	Types of surgical facility	Number of departments	Outcomes: bacterial count in site sampled air (CFU/m^3^), risk of SSI, reduction in SSI	Conclusion	p value	References
Friberg et al. (2001)	The Journal of hospital infection	Comparative study	5	30	Surgical area contamination—comparison with or without laminar air-flow	Facility without LAF	14/30	Bacterial count in site sampled air 8 CFU/m^3^	Use of horizontal LAF units is seriously questionable	< 0.001	[17]
						Facility with LAF	16/30	Bacterial count in site sampled air 22 CFU/m^3^			
Kakwani et al. (2007)	Injury	consecutive cohort-study	88	435	To study the effect of laminar air-flow on the outcomes of hemiarthroplasty	Facility without LAF	223/435	Bacterial count in site sampled air 0.45 CFU/m^3^	Laminar air-flow equipped theatres were recommended for hemiarthroplasty procedures	< 0.001	[18]
						Facility with LAF	212/435	Bacterial count in site sampled air 5.4 CFU/m^3^			
Brandt et al. (2008)	Annals of Surgery	Retrospective cohort-study	44	99,230	Effect of Operating Room Ventilation with Laminar Airflow on the Surgical Site Infection (SSI) Rate	Facility without LAF	31,573/99230	Risk of SSI 1.06	OR ventilation with laminar airflow showed no benefit and associated with a significantly higher risk for severe SSI after surgery	< 0.001	[19]
						Facility with LAF	67,707/99230	Risk of SSI 1.63			
Nilson et al. (2010)	The Journal of hospital infection	Comparative study	22	8550	Assessment of laminar air flow reduced infection during surgery	Facility without LAF	3256/8550	Bacterial count in site sampled air 45 CFU/m3	LAF is efficient for reducing infection in operating room	< 0.001	[20]
						Facility with LAF	4725/8550	Bacterial count in site sampled air 275 CFU/m3			
Sossai et al. (2011)	Journal of orthopaedics and traumatology	Comparative study	2	17	Effect of LAF unit in reducing the bacterial contamination	Facility without LAF	6/17	Bacterial count in site sampled air 23.5 CFU/m^3^	LAF unit not helps in reducing the bacterial contamination of the wound area	< 0.05	[21]
						Facility with LAF	11/17	Bacterial count in site sampled air 3.5 CFU/m3			
Hooper et al. (2011)	The Journal of bone and joint surgery	Retrospective study	50	36,826	Effect of laminar flow in reducing the infections after total hip and knee replacement surgery	Facility without LAF	14,730/36826	Risk of SSI 0.110	Deep infections were not reduced by using the laminar air flow	< 0.001	[22]
						Facility with LAF	9206/36826	Risk of SSI 0.082			
Bosanquet et al. (2013)	Annals of the Royal college of Surgeons of England	Retrospective study	45	170	Effect of Laminar flow in reducing the surgical site infections in patients after surgery	Facility without LAF	114/170	Reduction in Surgical site infections 17%	laminar flow not helps in reducing the incidences of SSIs in patients after surgery	< 0.05	[23]
						Facility with LAF	56/170	Reduction in Surgical site infections 7%			
Pinder et al. (2016)	The bone and joint journal	Observational study	19	803 065	an observational study to demonstrate whether laminar flow ventilation reduce the rate of infection	Facility without LAF	296,653/803065	Reduction in Surgical site infections 2.7%	Installation of laminar flow causes no change in the incidences of SSIs	< 0.05	[24]
						Facility with LAF	562,412/803065	Reduction in Surgical site infections 3.8%			
Wang et al. (2020)	Orthopedics	Original cohort investigation	2	6972	Association of Laminar Airflow with Infection during total arthroplasty	Facility without LAF	3027/6972	Reduction in Surgical site infection 0.4%	There is no benefit of LAF in operating rooms	< 0.05	[25]
						Facility with LAF	3945/6972	Reduction in Surgical site infection 0.5%			
Langvatan et al. (2020)	Journal of hospital infection	Original investigation	62	51,292	Assessment of operating room ventilation and the risk of infection after total hip arthroplasty	Facility without LAF	2046/4313	Risk of SSI 0.7	Chances of infection is less without LAF than with LAF	0.01	[26]
						Facility with LAF	2647/4313	Risk of SSI 0.9			

### Meta-analysis results

A meta-analysis was performed using RevMan and MedCalc software. The results are discussed below:

### Risk of bias assessment

**Fig. 2:**
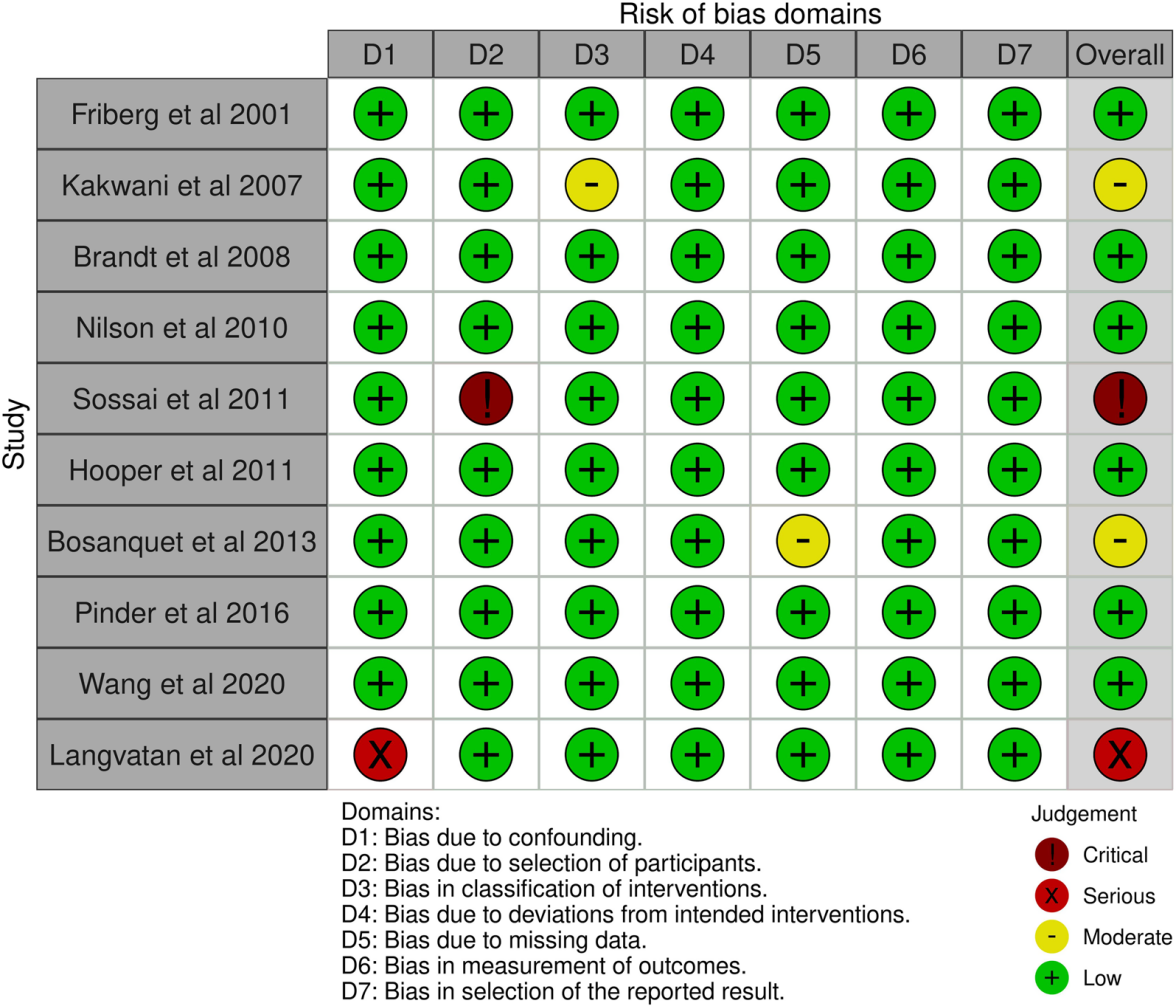
Risk bias summary

**Fig. 3:**
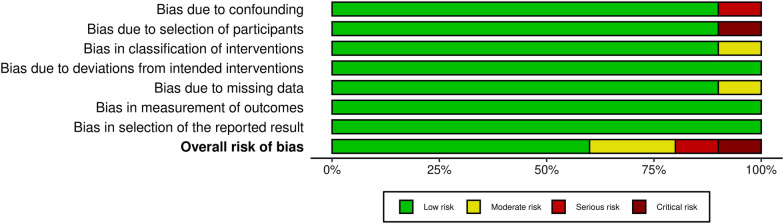
Risk of bias graph

**Fig. 4:**
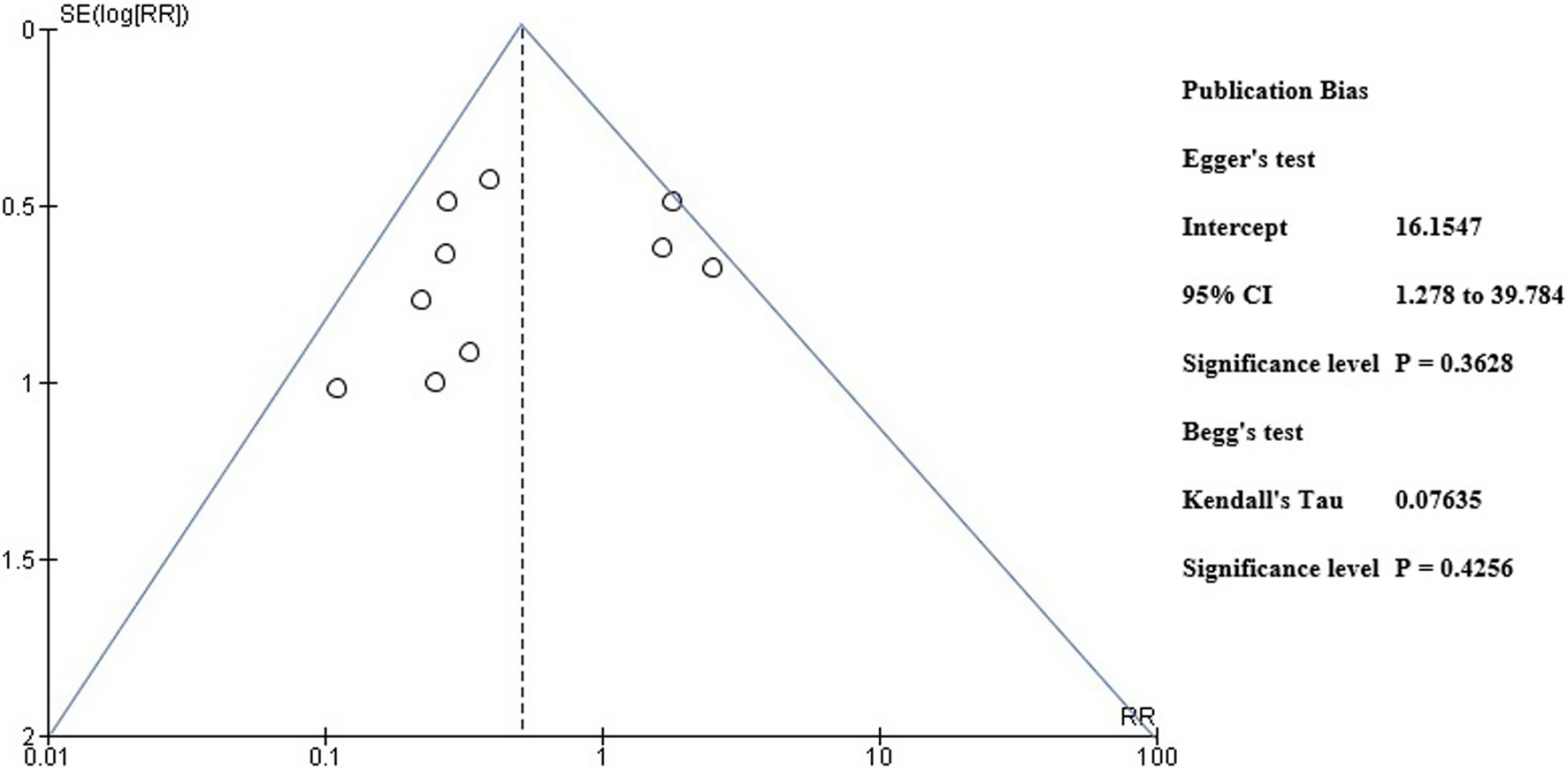
Funnel plot for publication bias

A pre-designed questionnaire was used for assessment of Risk of bias and results are shown in Table 3. Figure 2 depicts the risk of bias summary, whereas Fig. 3 depicts the risk of bias graph. Six of the ten included studies were associated with low risk of bias whereas two had a moderate risk attributable to bias in classification of intervention and bias due to missing data. One study posed a serious risk of bias due to confounding and one has critical risk of bias due to selection of participants. The current meta-analysis has a low risk of publication bias as apparent from the funnel plot shown in Fig. 4**,** and the p values of both tests are non-significant (p > 0.05) [32]. Egger’s test p-value is 0.3628 and Begg’s test p-value is 0.4256.

**Table 3:** Risk Assessment for Included Studies

	Friberg et al. [17]	Kakwani et al. [18]	Brandt et al. [19]	Nilson et al. [20]	Sossai et al. [21]	Hooper et al. [22]	Bosanquet et al. [23]	Pinder et al. [24]	Wang et al. [25]	Langvatan et al. [25]
Was a consecutive or random sample of patients enrolled?	Yes	Yes	Yes	Yes	Yes	Yes	YES	Yes	Yes	Yes
Did the studies avoid inappropriate exclusions	Yes	Yes	Yes	Yes	Yes	Yes	Yes	Yes	Yes	Yes
Did all patients receive the same reference standard	Yes	Yes	Yes	Yes	Yes	Yes	Yes	Yes	Yes	Yes
Were all patients included in the analysis	No	No	No	No	No	No	No	NO	NO	NO
Was the sample frame appropriate to address the target population?	Yes	Yes	Yes	Yes	Yes	Yes	Yes	Yes	Yes	Yes
Were studies participants sampled in an appropriate way?	Yes	Yes	Yes	Yes	Yes	Yes	Yes	Yes	Yes	Yes
Were the studies subjects and the setting described in detail?	Yes	Yes	Yes	Yes	Yes	Yes	Yes	Yes	Yes	Yes
Were valid methods used for the identification of the condition?	Yes	Yes	Yes	Yes	Yes	Yes	Yes	Yes	Yes	Yes
Was the condition measured in a standard, reliable ways for all participants?	Yes	Yes	Yes	Yes	Yes	Yes	Yes	Yes	Yes	Yes
Was there appropriate statistical analysis?	Yes	Yes	Yes	Yes	Yes	Yes	Yes	Yes	Yes	Yes

### Statistical assessment

**Fig. 5:**
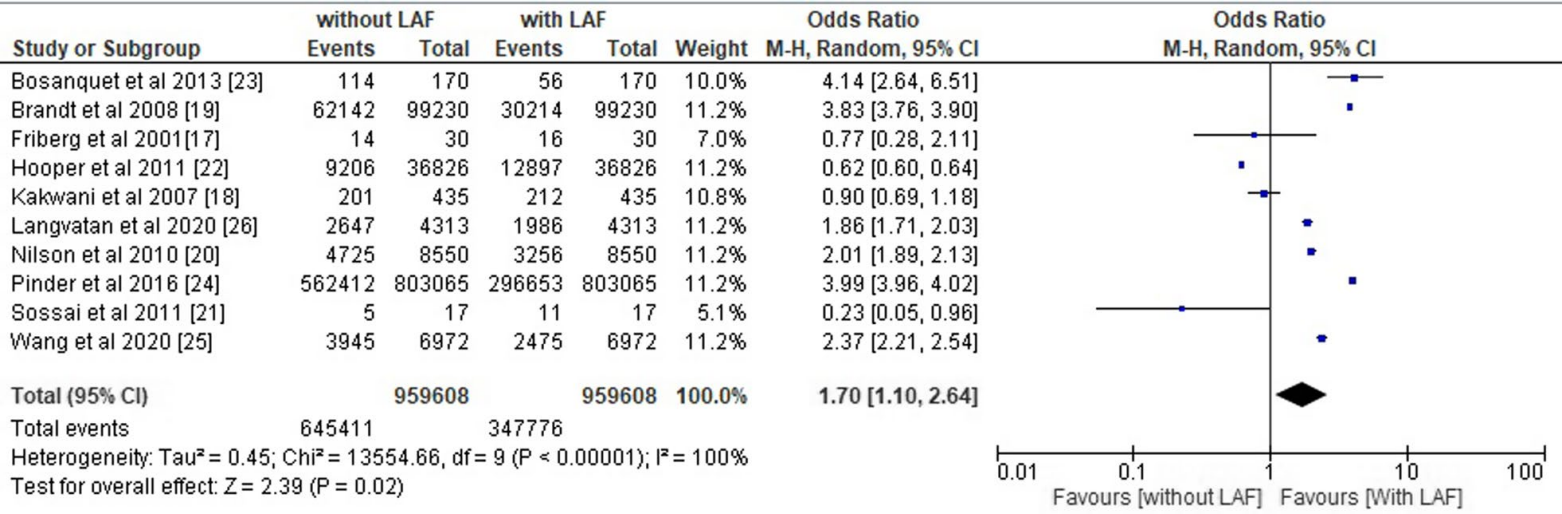
Forest plot for overall odds ratio of the included studies

**Fig. 6:**
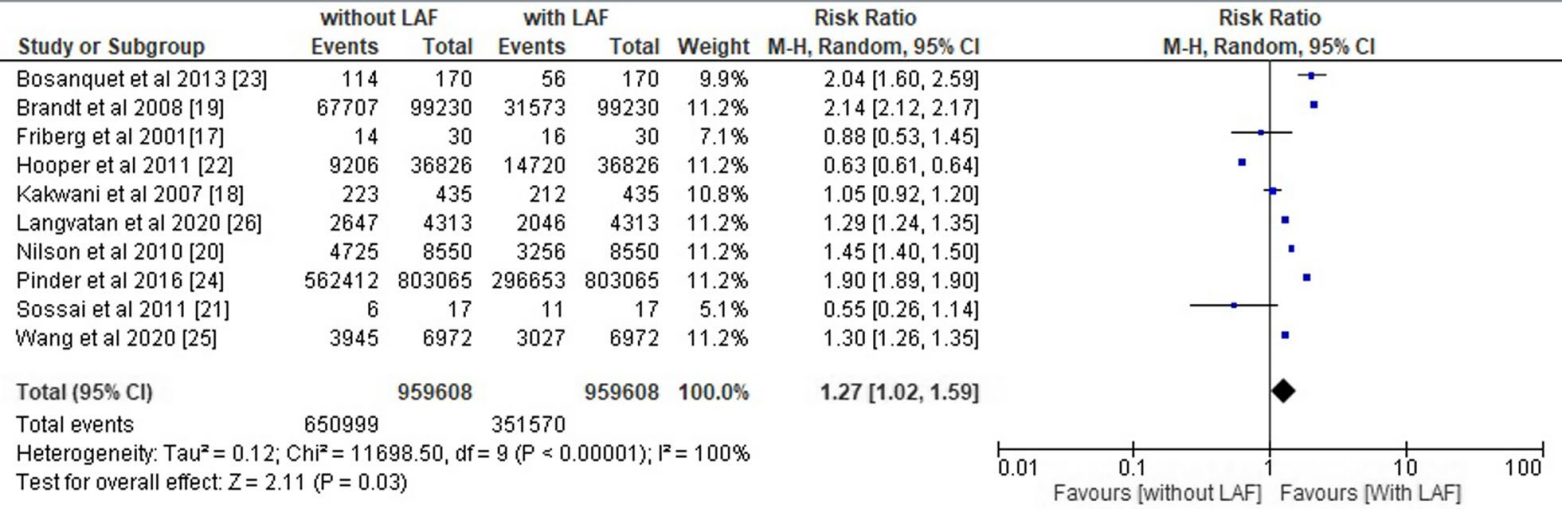
Forest plot for overall risk ratio of the included studies

The overall pooled odds ratio and risk ratio of the included studies were calculated using RevMan software and their respective forest plots were designed as shown in Figs. 5 and 6. We obtained the overall pooled odds ratio (OR) of all the included studies equals to 1.70 (95% CI 1.10–2.64) with heterogeneity of *Tau*^2^ 0.45, *chi*^2^ 13,554.66, df 9, I^2^ 100%, z value 2.39 and p < 0.05 and overall pooled risk ratio (RR) of 1.27 (95% CI 1.02–1.59) with heterogeneity of *Tau*^2^ 0.12, *chi*^2^ 11,698.50, df 9, I^2^ 100%, z value 2.11 and p < 0.05.

**Fig. 7:**
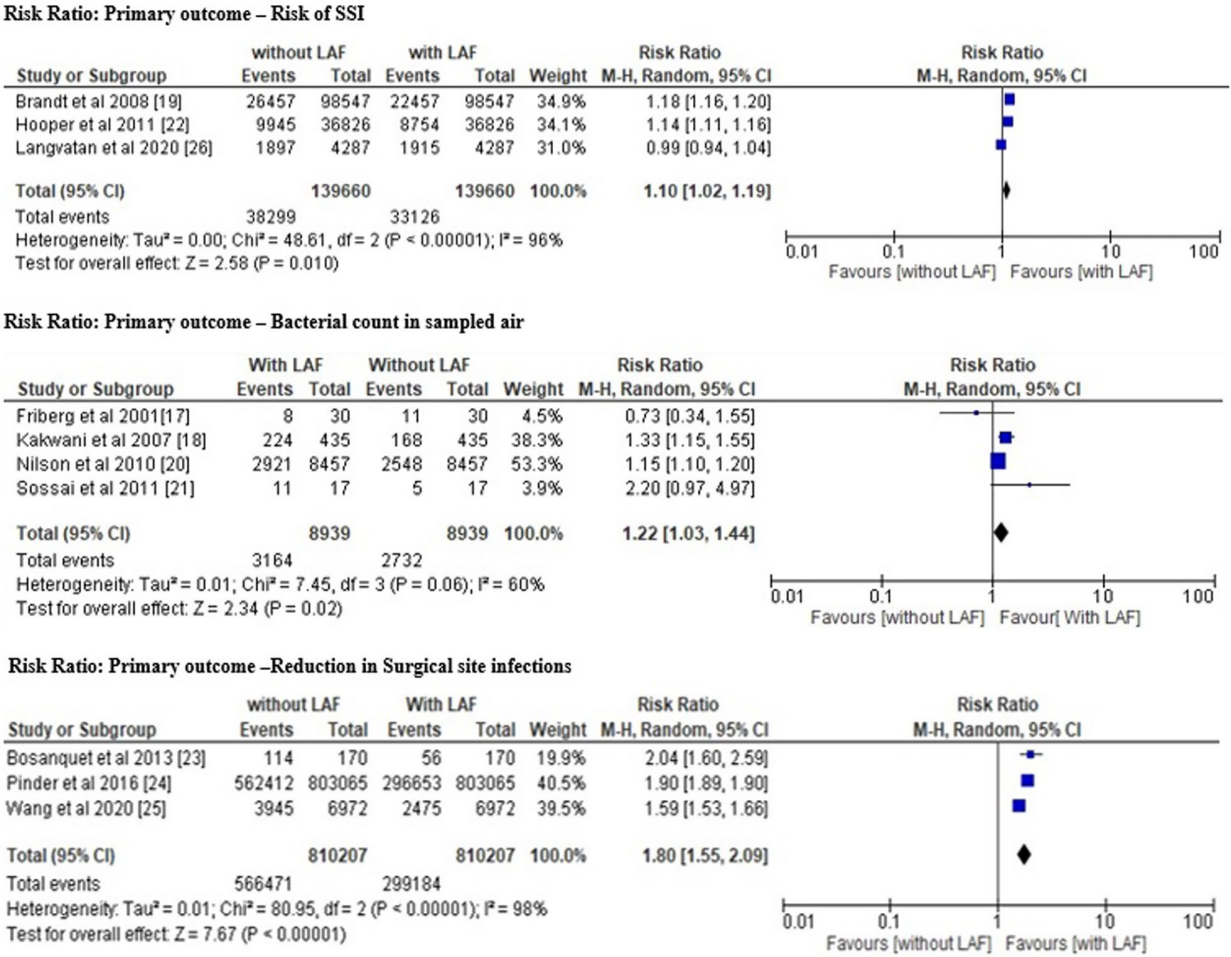
Forest plot risk ratio of different primary outcomes

The different primary outcomes of the included studies (risk of SSI, bacterial count in sampled air and reduction in SSI) were also assessed separately as shown in Fig. 7, 8 analyse the benefits of presence and absence of LAF as mentioned below:

**Fig. 8:**
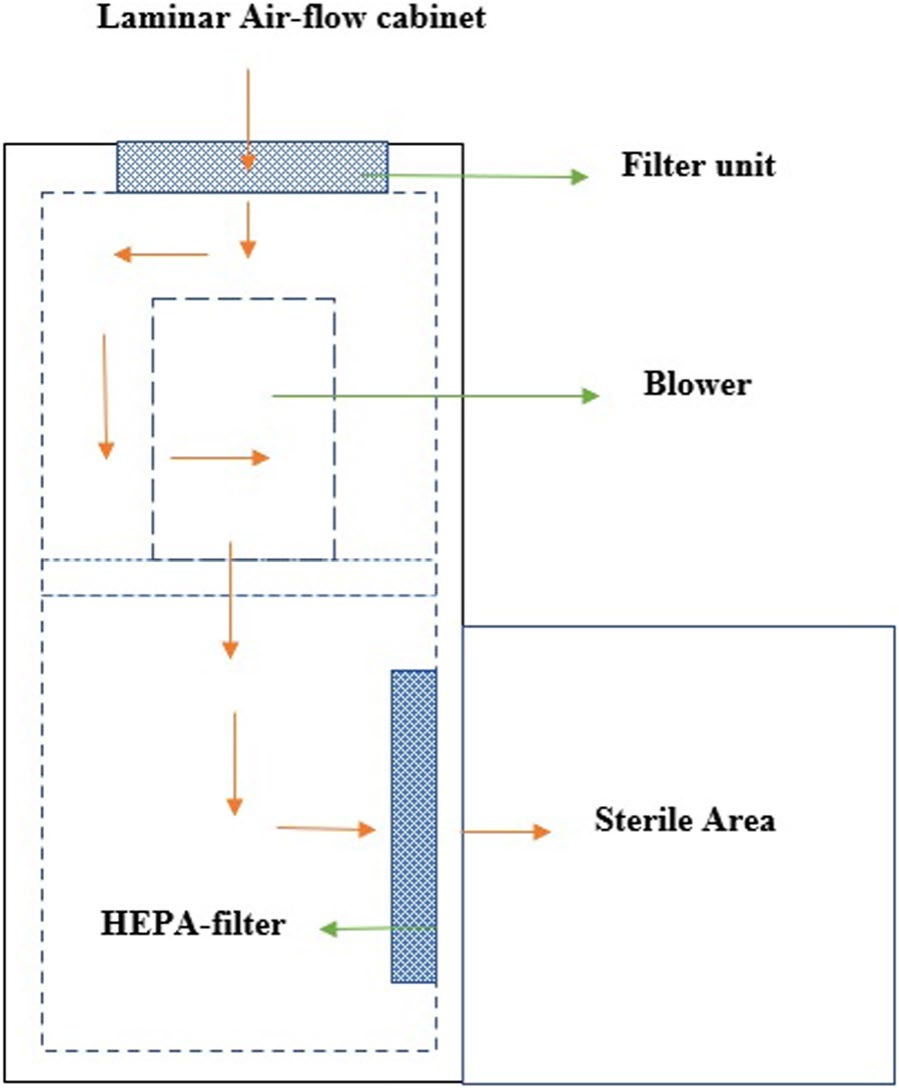
Design of Laminar air flow cabinet

*Results for primary outcome: risk of SSI* We obtained the pooled risk ratio of 1.10 [95%CI 1.02–1.19] with the heterogeneity of *Tau*^2^ 0.00, *chi*^2^ 48.61, df 2, I^2^ 96%, z value 2.58 and p = 0.01.

*Results for primary outcome: bacterial count in sampled air* We obtained the pooled risk ratio of 1.22 [95% CI 1.03–1.44] with the heterogeneity of *Tau*^2^ 0.01, *chi*^2^ 7.45, df 3, I^2^ 60%, z value 2.34 and p = 0.02.

*Results for primary outcome: reduction in SSI* We obtained the pooled risk ratio of 1.80 [95% CI 1.55–2.09] with the heterogeneity of *Tau*^2^ 0.01, *chi*^2^ 80.95, df 2, I^2^ 98%, z value 7.67 and p = 0.00001.

The risk ratio and odds ratio value greater than 1 indicates the high likelihood of contamination and inefficiency of LAF in the prevention of SSIs in operating room during orthopedic surgeries. All of these results are statistically significant with a p-value less than 0.05 [33–35] and indicate that the use of a LAF ventilation system in operating rooms is not worth reducing the chances of the prevention of SSIs.

## Discussion

SSIs pose a significant medical concern as patients are required to endure both the surgical procedures and the subsequent recovery period [36, 37]. Therefore, it is imperative to implement various measures to decrease the incidence of SSIs in the operating rooms. The utilization of sterile attire, bandages, and antiseptic cleansers has been shown to be beneficial [38–40]. It is recommended that the operating theatre be equipped with a LAF ventilation system.

A ventilation system that utilizes LAF is employed in operation theatres to produce air that is devoid of bacteria, thereby reducing the presence of infectious microorganisms in the air. The utilization of high-efficiency particle airflow in LAF systems results in the elimination of airborne pollutants and the establishment of a sterile environment. A sterile environment is essential for conducting microbiological experiments. The successful operation of the LAF chamber is contingent upon the utilization of a filter pad, fan, and high-efficiency particle air filter. The filter pad is designed to capture a majority of airborne pollutants, which are subsequently drawn in by a fan or blower. A high-efficiency particle air filter is capable of eliminating various types of airborne contaminants such as fungus spores, bacteria, and dust particles.

The results depicted in Fig. 8 indicate that the circulation of sterile air is evenly distributed throughout the workstation. [41, 42]. The operating room is equipped with an efficient air supply system, wherein the air is replaced at a rate of 15–25 times per hour. The air filtration systems utilized in operating rooms have been found to eliminate a significant proportion of particles that exceed 5 µm in size, with a range of 87–90%. LAF systems equipped with HEPA filters have the capacity to capture particles larger than 0.3 m with an efficiency of 99.97%. Despite their efficacy, these filters are associated with high costs that can impact the overall expenses of hospitals and surgical procedures, as reported in previous studies [43, 44].

The efficacy of using a surgical smoke evacuation system in preventing SSIs has been questioned by Kumin et al. [45] and Jain et al. [46] through their systematic review and meta-analysis. These studies have highlighted the high installation cost and the possibility of germs being trapped in the system's filter as potential drawbacks, despite other studies supporting its use. A higher incidence of infection was observed in orthopaedic operating rooms equipped with LAF systems. The pre-filter, also known as the filter pad, functions to purify the air prior to its entry into the cabinet, thereby facilitating a streamlined airflow. Subsequently, the fan facilitates the circulation of air towards the HEPA filters to undergo filtration.

HEPA filters are capable of capturing various types of particulate pollutants, such as bacteria and fungus, and subsequently emitting air that is free of particles. Failure to regularly clean these filters can result in the accumulation of germs, leading to the formation of microbial traps. Therefore, the extended usage of these items would not lead to a reduction in infection rates through the enhancement of air quality. Instead, it would result in the propagation of the disease and a heightened susceptibility to SSIs. Therefore, HEPA filters are not advisable owing to their elevated expenses and potential for contagion. In a similar vein, Sadrizadeh et al. [47] discovered that surgical garment systems used in operating rooms featuring LAF are a primary cause of post-operative infections, thereby restricting their usage. Similarly, Takutu et al. [48], Marasault et al. [49], Amiraslanpour et al. [50] have documented the constraints of the intervention and have not made any reference to its implementation in the surgical theater.

In line with these studies, our meta-analysis also revealed a pooled odds ratio (OR) value of 1.64 (95% CI 1.23–2.20) and a pooled risk ratio value of 1.30 (95% CI 1.14–1.48). The odds ratio and risk ratio values are greater than 1, which indicates a significant possibility that laminar airflow will increase the likelihood of SSIs rather than decrease them. The risk ratio of all the primary outcomes, including the risk of SSIs, the bacterial count in sample air, and the decrease in SSI incidences in patients in operating rooms with LAF installed, was also greater than 1, favouring the likelihood of higher infection risk in operating rooms with LAF. These findings supported the conclusion of the meta-analysis that LAF ventilation systems are not beneficial to patients undergoing orthopaedic surgery and should not be deployed in operating rooms.

### Limitations

The limitation of the present study is that the here only English language articles were included which can cause possible bias in the paper selection. Other than this, evaluation of parameters via different scales also influences the result upto some extent. Data of other relevant studies that mentions the proper documentation regarding the case history of patient’s, clinical issues can also be included to assess the details about the pre-and post-operative patients’ health status to indicate the importance and efficiency of these studies more clearly and estimating the efficacy of LAF in reducing SSIs in patients undergoing surgery.

## Conclusion

It's an urgent medical concern to find suitable ways to reduce surgical site infections for the successful recovery of patients after surgery and to reduce the time of hospital stay. Although for this purpose, the use of a LAF ventilation system is suggested, owing to its high installation cost and chances of microbial traps in filters that can enhance the infection instead of reducing it, it is of limited use. Based on our systematic review and statistically significant meta-analysis, we also found it ineffective in reducing SSIs after orthopedic surgeries and therefore advise against installing it in the operating room to save both the patient's health and money.

## Data Availability

The datasets used and/or analysed during the current study are available from the corresponding author on reasonable request.
